# Does clinical outcome of birch pollen immunotherapy relate to induction of blocking antibodies preventing IgE from allergen binding? A pilot study monitoring responses during first year of AIT

**DOI:** 10.1186/s13601-018-0226-7

**Published:** 2018-10-08

**Authors:** Sara Huber, Roland Lang, Markus Steiner, Lorenz Aglas, Fatima Ferreira, Michael Wallner, Thomas Hawranek, Gabriele Gadermaier

**Affiliations:** 1 Department of Biosciences, University of Salzburg, Hellbrunnerstraße 34, 5020 Salzburg, Austria; 2 Department of Dermatology, Paracelsus Medical University Salzburg, Salzburg, Austria; 3 Department of Internal Medicine III with Hematology, Medical Oncology, Hemostaseology, Infectious Disease, Rheumatology, Oncologic Center, Laboratory for Immunological and Molecular Cancer Research, Paracelsus Medical University Salzburg, Salzburg, Austria

**Keywords:** Birch pollen, Blocking capacity, Immunotherapy, Bet v 1, IgE, FAB assay, Inhibition mediator release assay, Allergens and epitopes

## Abstract

**Background:**

The clinical benefit of allergen-specific immunotherapy (AIT) involves induction of blocking antibodies. It is not clear if these antibodies function via steric hindrance alone or a combination of levels, avidities, and epitope specificities, and clinical outcome cannot be predicted. We aim to in-depth characterize serum antibody profiles during birch pollen AIT, investigate therapy-induced antibodies for their capacity to block IgE binding to Bet v 1 and correlate data with clinical outcomes.

**Methods:**

Immune responses of five birch pollen allergic patients were monitored during the first year of AIT by nasal provocation tests (NPTs), ImmunoCAP, immunoblots, direct and avidity enzyme-linked immunosorbent assays, mediator release assays, facilitated antigen binding (FAB) assays, and inhibition mediator release assays.

**Results:**

There was no correlation between NPT results and therapy-induced changes in levels (IgE, IgG, IgA, IgM), avidities, or mediator release potency of Bet v 1-specific antibodies. In FAB assays, blocking antibodies initiated upon AIT were shown to prevent formation of Bet v 1-IgE complexes of an indicator serum pool and significantly correlated with clinical readout. Inhibition mediator release assays using patient-specific IgE for passive sensitization revealed therapy-induced blocking capacities with very good correlation to NPT results. Notably, this assay was the only one to detect a non-responder during treatment in this pilot study.

**Conclusions:**

Clinical outcome of AIT depends on induction of blocking antibodies able to prevent the patient’s own IgE from allergen binding. Monitoring of clinical efficacy seems to be best achieved using the inhibition mediator release assay, as development of relevant blocking antibodies can be verified in a patient-tailored manner.

**Electronic supplementary material:**

The online version of this article (10.1186/s13601-018-0226-7) contains supplementary material, which is available to authorized users.

## Background

The enormous global increase in allergic diseases over the last few decades has become a major public health concern [1]. Having already reached epidemic proportions, the prevalence of allergic diseases is suspected to be still rising [2]. Currently, the only available curative treatment is AIT, which not only alleviates allergy symptoms, but also leads to a long-lasting clinical benefit by targeting the underlying immune response [3]. A hallmark of AIT is the induction of blocking antibodies, especially IgG4 and to some extent IgA [4–6]. Due to the sharing of epitopes, these antibodies can prevent IgE antibodies from allergen binding and in turn from receptor cross-linking [7]. This leads to a reduction in mediator release from mast cells and basophils, which is associated with allergic symptoms [8]. Although some general mechanisms have been elucidated, there is large interpatient variability in terms of AIT outcome, which turns monitoring of the course and effectiveness into a major challenge [9]. Consensus on objective monitoring of AIT progress would be useful to identify non-responders prior to treatment end, and would represent a clear benefit for patients and the overall health system.

At the B cell level, serum IgE levels are initially boosted by AIT but then decline during treatment. However, the decrease occurs too long after the start of treatment to be associated with clinical efficacy. Induction of high levels of serum IgG (especially IgG1 and IgG4) during AIT does not correlate with IgE reduction or treatment efficacy [10, 11]. Some studies suggest (1) allergen-specific IgE/total IgE [12], (2) allergen-specific IgG4/IgG1 [13], or (3) allergen-specific IgE/IgG4 [14] as more promising candidates for outcome prediction, but others were not able to verify these data [15–17]. Allergen-specific serum IgA showed either increased or unaltered levels during AIT, while no correlation with clinical outcome was observed [18–21]. The few studies dealing with allergen-specific IgM reported relatively unaltered levels [22]. Thus, quantities of serum antibody subclasses are not suitable for monitoring the clinical success of AIT [11]. A more detailed evaluation of antibody responses and their therapy-related changes is required.

Recent studies indicate that not only serum antibody subclass levels but rather their functional roles are important for successful therapy. The ability of IgG4 to block IgE-allergen binding was explained by the development of similar epitope specificities [7, 23]. Antibody affinities might also influence therapy outcome and increased somatic hypermutations has been reported to result in enhanced IgG affinity [24–27]. It remains to be investigated whether the AIT-induced blocking capacity solely results from steric hindrance or from a combined effect including functional antibody activity (hereafter termed antibody quality). Here, we focused on birch pollen allergy, one of the main causes for rhino-conjunctivitis in North America and Northern and Central Europe with a total incidence of more than 100 million people [28]. Although pollen of the white birch *Betula verrucosa* contains a complex mixture of proteins, more than 95% of birch pollen allergic patients are sensitized to the major allergen Bet v 1 and 60% demonstrate exclusive reactivity [29]. The potent allergenicity of Bet v 1 and the reported increasing prevalence, as well as socio-economic burden, warrants development of novel diagnostics, therapeutics, and AIT biomarkers.

In this pilot study, we extensively characterized Bet v 1 serum antibody responses during the early course of AIT in five birch pollen allergic patients. Antibody subclasses were analysed regarding levels, avidity, epitope specificities, and blocking capacities and correlated with rhino-conjunctivitis total symptom scores (RTSS) throughout the study. By integrating quantity and quality, as well as AIT-induced alterations of antibody-allergen interactions, we aimed to explain the therapy-related antibody blocking concept in more detail, and to monitor the allergic state of the patients throughout the first year of treatment.

## Methods

### Patients

Birch pollen-allergic patients qualifying for AIT in the routine clinical setting were selected according to case history, positive in vivo as well as in vitro diagnosis (n = 5) (Table 1). Inclusion criteria were a clinical history of moderate to severe birch pollen allergy (≥ 2 years), positive SPT towards birch pollen, Bet v 1 ImmunoCAP ≥ 3 (Phadia/Thermo Fisher Scientific, Uppsala, Sweden), and no history of AIT. Serum from a non-allergic (NA) and a non-birch pollen allergic (NBA) donor with negative SPT and IgE ImmunoCAP to birch were included as controls. Experiments using anonymized serum samples were approved by the local ethics committee of Salzburg (No. 415-E/1398/4-2011). Informed written consent was obtained from all study participants.

**Table 1:** Patients’ table

Serum donor	Age (years)	Sex	Birch pollen					ImmunoCAP			Other sensitizations
			Allergy symptoms	Skin prick test	Rhino-conjunctivitis total symptom scores			Bet v 1-specific IgE (kU/l)			Skin prick test
					T0	T1	T2	T0	T1	T2	
P1	41	m	RC	+++	6	2	1	40.2	42.7	19.8	Mites, hazel pollen
P2	50	m	RC	+++	8	1	1	4.4	5.8	4.8	Grass, hazel pollen
P3	54	m	RC	+	3	0	2	26.3	53.3	38.0	Mites, hazel pollen
P4	45	m	RC	++	3	2	0	51.1	159.0	81.3	Mold, hazel pollen
P5	63	f	RC	+	4	3	1	10.3	17.5	10.0	Mugwort, ragweed, hazel pollen
NA	47	f	None	Neg				< 0.01			None
NBA	26	f	None	Neg				< 0.01			Mites

P1–5, AIT patients; NA, non-allergic serum donor; NBA, non-birch allergic serum donor; RC, rhino-conjunctivitis; +, mild reaction; ++, moderate reaction; +++, strong reaction; Neg, negative; T0, before AIT; T1, 2 weeks after reaching the maintenance dose; T2, 1 year after starting AIT

### AIT and in vitro IgE reactivity

**Fig. 1:**
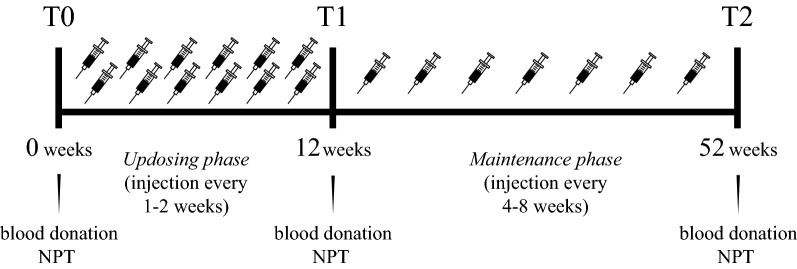
AIT timeline and study design. Subcutaneous immunotherapy with birch pollen extract was performed with 11–13 injections at intervals of 1–2 weeks during the updosing phase. After reaching the maintenance dose, injections were further administered every 4–8 weeks until 52 weeks. Serum samples were obtained before treatment (T0), 2 weeks after reaching maintenance dose (T1), and 1 year after starting treatment (T2). At the same time points, NPT were performed and RTSS were noted

Subcutaneous immunotherapy was performed using Alutard SQ Birch (ALK-Abelló, Hørsholm, Denmark) with 11–13 injections at intervals of 1–2 weeks during the up-dosing phase starting with 100 SQ according to the manufacturer’s protocol (Fig. 1). After reaching 100,000 SQ maintenance dose (corresponding to 12.3 µg Bet v 1), injections were further administered every 4–8 weeks until 1 year. Serum samples were obtained before treatment (T0), 2 weeks after reaching maintenance dose (T1), and 1 year after starting treatment (T2). Bet v 1-specific IgE and IgG4 (t215), Bet v 2 (t216), Bet v 4 (t220) and total IgE (t3) was determined using ImmunoCAP (Thermo Fisher Scientific) and is reported in Additional file 1.

### Nasal provocation test (NPT)

Nasal provocation tests were performed with a commercially available birch pollen extract (AllergoPharma, Reinbek, Germany) reconstituted in 5 ml AllergoPharma solvent. If negative to saline challenge, NPT was performed with increasing concentrations of birch pollen extract (5-5000 SBE/ml, 23.1 µg Bet v 1 in 5000 SBE) at 15-min intervals (150 µl per puff, at 5000SBE/ml 2 puffs). Sneezing, rhinorrhea, nasal pruritus, nasal obstruction and congestion, as well as ocular symptoms were recorded and a RTSS ranging from 0 to 18 was established, where each symptom was assessed on a four-point scale (0 = absent, 1 = mild, 2 = moderate, 3 = severe) according to published protocols [30].

### Preparation of birch pollen extract and recombinant Bet v 1

Aqueous pollen extract was prepared by rapid agitation of 2 mg birch pollen (Allergon AB, Ängelholm, Sweden) dissolved in 10 ml distilled water for 4 h at room temperature. After centrifugation and 0.45 µm filtration, the protein concentration was measured by Bradford assay (Pierce Coomassie Protein Assay Kit, Thermo Scientific, Rockford, IL, USA) and Bet v 1 concentration determined as 0.76 mg/ml by a sandwich ELISA. Production and characterization of recombinant Bet v 1.0101 was performed according to published methods [31]. In short, the allergen was produced as non-fusion protein in *E. coli* BL21 Star (DE3) using the pET28b vector system and purified from the soluble fraction by standard chromatographic procedures. The endotoxin level determined by limulus amoebocyte lysate cell assay was < 0.3 ng/ml.

### Immunoblot

Birch pollen extracts were separated by preparative 15% polyacrylamide gels and blotted onto nitrocellulose membranes (Whatman, Maidstone, UK). Blots were blocked for 1 h with a buffer containing 25 mM Tris/Cl, pH 7.5, 150 mM NaCl, 0.5% (v/v) Tween, 0.5% (w/v) BSA, 0.05% (w/v) NaN_3_ and cut into stripes which were incubated with 1:10 diluted sera overnight at 4 °C while shaking. Bound allergen-specific IgE, IgG1, IgG4, IgA, as well as IgM was detected using AP-conjugated monoclonal anti-human IgE (BD Biosciences, Franklin Lakes, NJ, USA; diluted 1:10,000 in blocking buffer), IgG1, IgG4, IgA or IgM antibodies (all Southern Biotech, Birmingham, AL, USA; diluted 1:10,000 in blocking buffer), respectively. Furthermore, a staining solution consisting of 10 ml AP buffer (100 mM Tris/Cl, pH 9.5, 100 mM NaCl, 5 mM MgCl_2_), 30 µl BCIP (stock: 50 mg/ml in dimethylformamide) and 30 µl NBT (stock: 100 mg/ml in 70% dimethylformamide, 30% sterile water) was used. After staining, membranes were washed with distilled water and air-dried.

### Direct and avidity enzyme-linked immunosorbent assay (ELISA)

For direct ELISA experiments, Maxisorp plates (Nunc, Thermo Fisher, Waltham, MA, USA) were coated with 100 ng Bet v 1/well in PBS overnight at 4 °C. After washing with TBS, pH 7.4, 0.05% (v/v) Tween, plates were blocked with TBS, pH 7.4, 0.05% (v/v) Tween, 0.5% (w/v) BSA for 1 h at room temperature. They were either incubated with serial dilutions of sera (1:10 for IgE, IgG1 and IgG4 detection, 1:40 for IgA and IgM detection) for endpoint titer determination or with just one dilution of patients’ sera (basically the factor was chosen between an OD_405/492_ of 0.5 and 1 where the titration curves were not in the plateau; 1:10 for IgE, 1:40 for IgG4 and IgG1, 1:160 for IgA and IgM) for detection of the reactivity of antibody subclasses before and after heat inactivation overnight at 4 °C. Bound allergen-specific immunoglobulins were detected with alkaline phosphatase (AP)-conjugated monoclonal anti-human IgE (BD Biosciences, Franklin Lakes, NJ, USA), IgG1, IgG4, IgA or IgM antibodies (Southern Biotech, Birmingham, AL, USA), respectively, after incubation for 1 h at 37 °C and for 1 h at 4 °C. For colorimetric detection 10 mM PNPP (Applichem GmbH, Darmstadt, Germany) was used as substrate and OD measurements were performed on a Tecan Sunrise (Tecan Group LTd., Männedorf, Switzerland) plate reader at 405/492 nm. Measurements were performed as duplicates and results are shown as mean values. For calculation of endpoint titer, the LOD (Limit of Detection) was used as cutoff value which is calculated as three times the standard deviation.

For avidity ELISA experiments, Bet v 1 was coated on Maxisorp plates and washed as indicated above. 50 µl diluted sera were pipetted in duplicates into 12 wells each (same amount of serum in each well). The serum dilution for each serum was chosen between an OD_405/492_ of 0.5 and 1 where the titration curves of the direct ELISA were not in the plateau). After an incubation step overnight at 4 °C, 50 µl/well sodium thiocyanate (NaSCN) (Applichem) solution was added to the wells in concentrations ranging from 0 M to 3 M NaSCN (100% value, no inhibition) and plates left at room temperature for 15 min. Bound allergen-specific IgE, IgG1, IgG4, IgA or IgM was detected with AP-conjugated monoclonal antibodies and a coloritmetric substrate as indicated above. Avidity indices were calculated from the NaSCN-concentration at which 50% of the bound antibodies were detached. Measurements were performed as duplicates and mean values are given.

### FAB assay

The capacity of birch pollen AIT-induced antibodies to block IgE antibody binding to Bet v 1 was monitored using a FAB assay which was performed according to slightly modified published methods [32, 33]. In short, a pool of indicator sera (20 µl, all RAST class 6 towards Bet v 1) was combined with 15 µl of sera termed test sera in the following (or EBV medium for control samples to obtain 100% complex formation) and 5 µl of allergen solution in the appropriate concentration (for Bet v 1: 0.1 µg/ml). To allow IgE-allergen complex formation this mix was incubated for 1 h at 37 °C. Thereafter, 1 × 10^5^ EBV transformed B-cells (in 5 µl) were added for 1 h at 4 °C. IgE complexes bound to the low affinity IgE receptor FcεRII on B-cells were stained with FITC-labelled affinity purified goat-anti human IgE antibody (KPL, Gaithersburg, MD, USA) and measured on a FACS Canto II flow cytometer (Becton, Dickinson and Company, Franklin Lakes, New Jersey, USA). The blocking capacity of AIT-induced antibodies was expressed as 100% minus percentage of cells carrying IgE-allergen complexes.

### Mediator release and inhibition mediator release assay

Mediator release assays were performed using RBL-2H3 rat basophilic leukemia cells transfected with the alpha chain of the human IgE receptor FcεRI, which were passively sensitized with the allergic patients’ sera. Sera were diluted 1:20 in tissue culture medium and incubated overnight at 37 °C and 7% CO_2_ [34]. After several washing steps, serial dilutions of Bet v 1 in Tyrode’s buffer (Sigma) supplemented with 1 g/L sodium bicarbonate, 0.1% (w/v) BSA, and 50% (v/v) deuterium oxide (Sigma) were added, leading to antigen-dependent β-hexosaminidase release into the supernatant. This was measured by enzymatic cleavage of the fluorogenic substrate 4-methylumbelliferyl-N-acetyl-β-glucosaminide (Sigma) after 1 h of incubation at 37 °C and expressed as percent of total enzyme content of Triton X-100 treated cells.

Inhibition RBL assays were performed according to published methods [22] with minor modifications. For inhibition RBL assays simulating FAB settings, RBL-2H3 rat basophilic leukemia cells were passively sensitized with IgE from the FAB indicator serum pool at a final dilution of 1:200 in tissue culture medium (dilution factor was adapted to the IgE endpoint titer of patients’ T0 sera and the indicator serum pool to use similar amounts of IgE) overnight. For inhibition, serial dilutions of T0, T1, and T2 sera obtained from the five AIT patients were added. These sera were heat-inactivated for 1 h at 56 °C to abolish IgE reactivity before they were pre-incubated with 10 ng/ml of Bet v 1 (depending on mediator release with indicator serum pool) and added to the sensitized cells.

For inhibition RBL assays used to monitor the AIT-induced blocking antibody responses on a patient-by patient basis, rat basophilic leukemia cells were passively sensitized with IgE from T0 serum samples at a final dilution of 1:20 in tissue culture medium overnight and the assay was performed as described above. Supernatants were analyzed for remaining β-hexosaminidase release. Results are expressed as percentage of inhibition in relation to the total β-hexosaminidase release obtained by addition of 10% Triton X-100. For statistical analysis, curve-representative inhibition values at inhibition serum dilutions of 1:10 were chosen.

### Statistical analysis

GraphPad Prism software 5 was used for statistical analysis. Friedman test with Dunns post test was used for comparison of the three AIT time points. *P* values < .05 were considered statistically significant. Pearson’s correlation of antibody and clinical data was calculated and displayed by regression lines. Pearson r (rho) values of ≥ 0.5 or ≤ − 0.5 were considered statistically significant.

## Results

### Clinical and serological profiles of AIT patients

In this pilot study, the antibody response of five birch pollen allergic patients (P1–P5; 4 males and 1 female, aged 41–63 years) undergoing AIT was monitored (Fig. 1). Patients suffered from rhino-conjunctivitis, showed positive SPT to birch pollen, and specific IgE to Bet v 1 (4.4–51.1 kU/L) prior to treatment (Table 1). A considerable decrease in RTSS towards birch was noted in four out of five patients in the course of AIT. For P3, RTSS initially decreased but returned almost to the pre-treatment value, suggesting no or low clinical benefit after 1 year treatment. As expected, Bet v 1-specific IgE levels initially increased and subsequently declined while IgG4 levels considerably increased during AIT (Additional file 1). Sensitization to Bet v 2 (profilin) and Bet v 4 (polcalcin) was negative with the exception of P4 demonstrating very low IgE reactivity to profilin after AIT (Additional file 1).

### Alterations in patients’ IgE and IgG reactivity mostly affect the major allergen Bet v 1

**Fig. 2:**
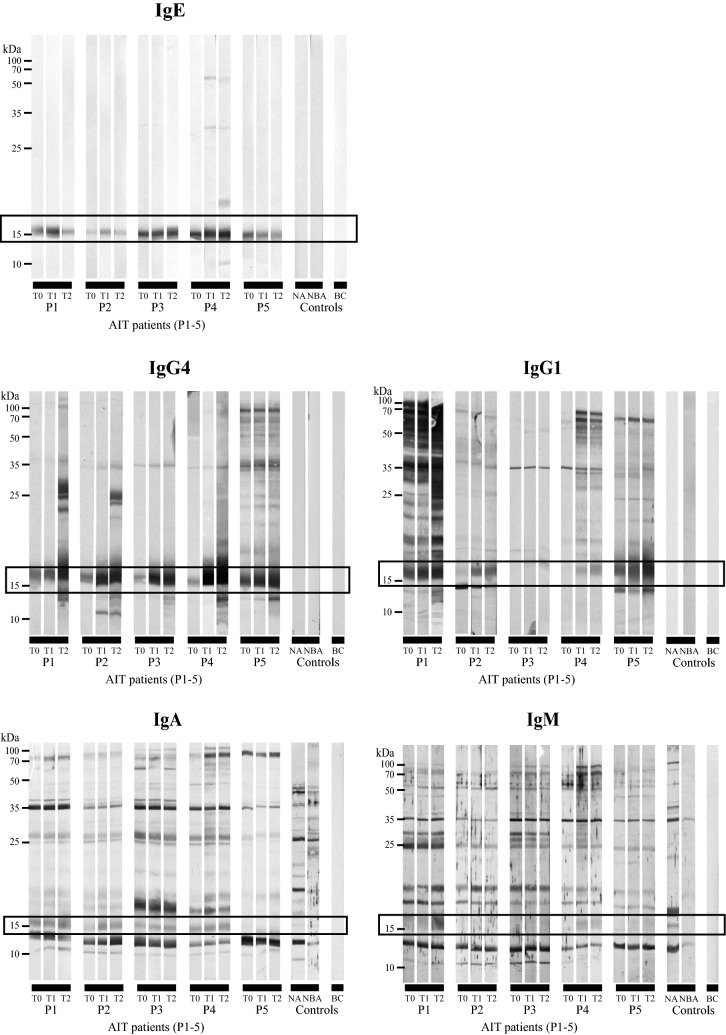
Reactivity of different antibody subclasses against birch pollen proteins and therapy-related changes. Immunoblot experiments with birch pollen extract and sera of birch pollen AIT patients (P1–5) collected at three time points of immunotherapy (T0, T1, and T2). Bet v 1 is framed in black; NA, non-allergic serum; NBA, non-birch allergic serum; BC, buffer control

To monitor therapy-related changes of antibody profiles towards whole birch pollen extract, we performed immunoblot experiments (Fig. 2). Patients’ IgE almost exclusively reacted against Bet v 1, only P4 developed low levels of novel IgE towards four additional birch pollen proteins. Serum IgG4 and IgG1 reactivity against Bet v 1 considerably increased over time except for P3 where IgG1 levels remained low. IgG recognition of other birch pollen proteins displayed a complex reactivity pattern and AIT-induced de novo sensitizations were rather frequent. By contrast, the highly diverse serum IgA and IgM profiles remained mostly unaffected and Bet v 1 levels were low and stable. Immunoblots with control sera revealed no IgE, IgG4 or IgG1 but IgA and IgM reactivity towards birch pollen proteins.

### Antibody subclass titer do not correlate with clinical efficacy of AIT

**Fig. 3:**
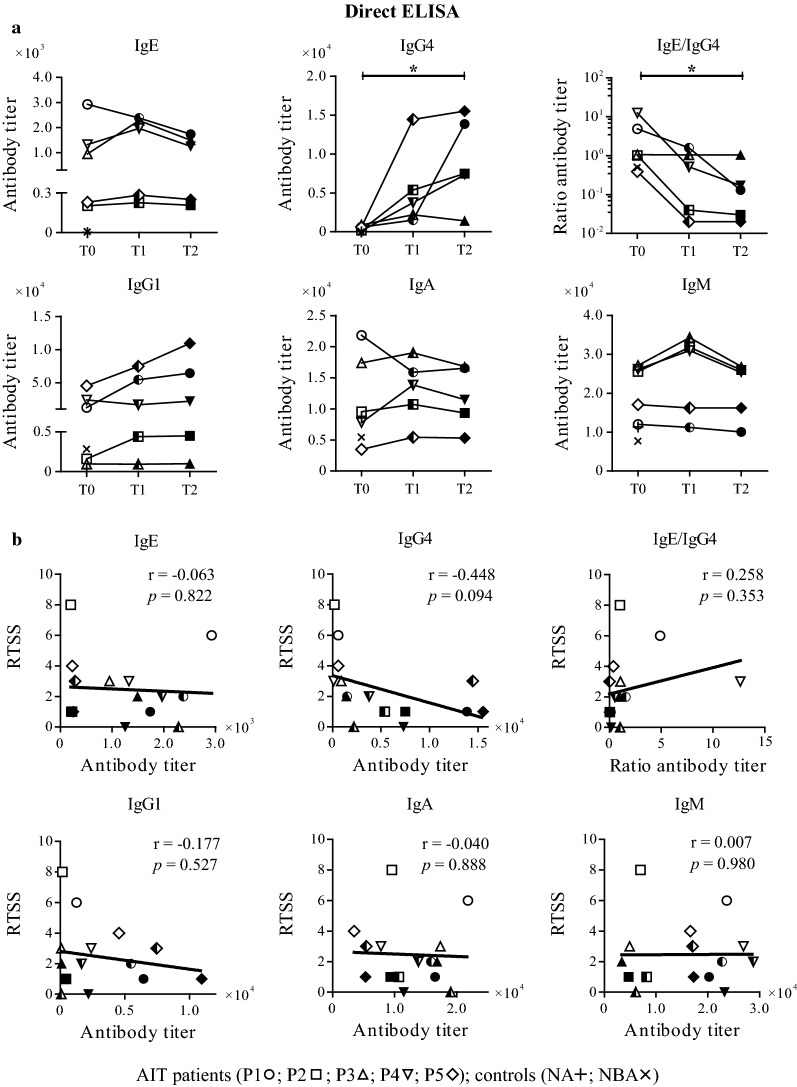
Bet v 1-specific serum antibody titer during AIT and in control sera analyzed by ELISA. **a** Therapy-related changes in antibody subclass titer. Statistics were performed with Friedman and Dunn’s post test. *p < 0.05. **b** Correlation of antibody titer with RTSS. Serum samples were obtained at three different time points (T0, open; T1, semi-filled; T2, filled symbols)

Since substantial therapy-related changes towards Bet v 1 were revealed by immunoblot, subsequent experiments focused on this clinically relevant allergen. First, we determined antibody titer by ELISA using individual sera (Fig. 3a, Additional file 2). Four out of five patients showed slightly enhanced Bet v 1-specific IgE at T1, followed by a decline at T2, while IgE levels of P1 decreased steadily over time (Fig. 3a). These results are in agreement with ImmunoCAP measurements (Additional file 3A). IgG4 levels significantly increased during therapy, showing an initial boost and further enhancement in four patients. By contrast, P3 showed a rather weak IgG4 increase, which was followed by a decline at T2. Considering IgE/IgG4 ratios, significant decreases were observed during AIT with the exception of P3, who presented unaltered ratios. Those results are coherent with ImmunoCAP results demonstrating highly significant correlation values (Additional file 3A). Three patients showed a steady increase in IgG1, while two patients including P3 had unaltered IgG1 levels during AIT. IgA and IgM levels were more or less unaltered with a minor increase at T1. In summary, neither antibody titers nor IgE/IgG4 ratios correlated with RTSS (Fig. 3b, Additional file 3B). Control sera revealed insignificant amounts for Bet v 1-specific IgE, IgG4 and IgG1, while IgA and IgM titers were similar to those of AIT patients (Additional file 2).

### The allergenic potential of patient sera during AIT is determined by IgE levels

**Fig. 4:**
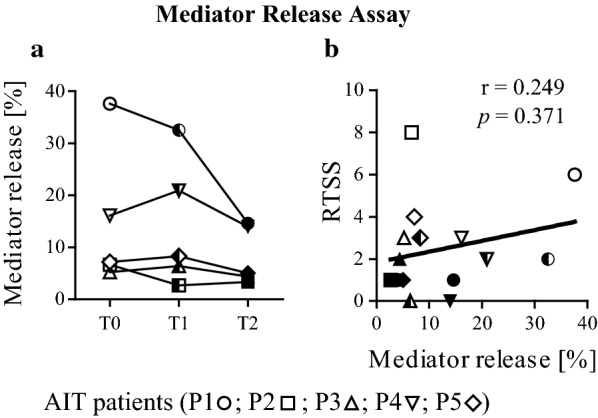
Bet v 1-specific β-hexosaminidase release during AIT analyzed by mediator release assays. **a** Therapy-related changes in mediator release triggered by 10 ng/ml Bet v 1. Statistics were performed with Friedman and Dunn’s post test. **b** Correlation of mediator release with RTSS. Serum samples were obtained at three different time points (T0, open; T1, semi-filled; T2, filled symbols)

We performed mediator release assays to determine the allergenic activity of patients’ sera triggered by IgE receptor cross-linking and subsequent β-hexosaminidase release (Additional file 4). Mediator release curves at a representative concentration of 10 ng/ml Bet v 1 were plotted for each sample (Fig. 4a). During AIT, the allergenic activity initially either decreased (P1, P2) or slightly increased (P3, P4, P5) and subsequently returned at least to basic values. Mediator release values correlated with IgE titer determined by ELISA while IgE ImmunoCAP values did not (Additional file 3C). Also, IgM titers showed correlating values (Additional file 4). Mediator release triggered by 10 ng/ml Bet v 1 did however not relate to RTSS results (Fig. 4b).

### Different antibody subclasses have heterogeneous patterns of avidity for Bet v 1

**Fig. 5:**
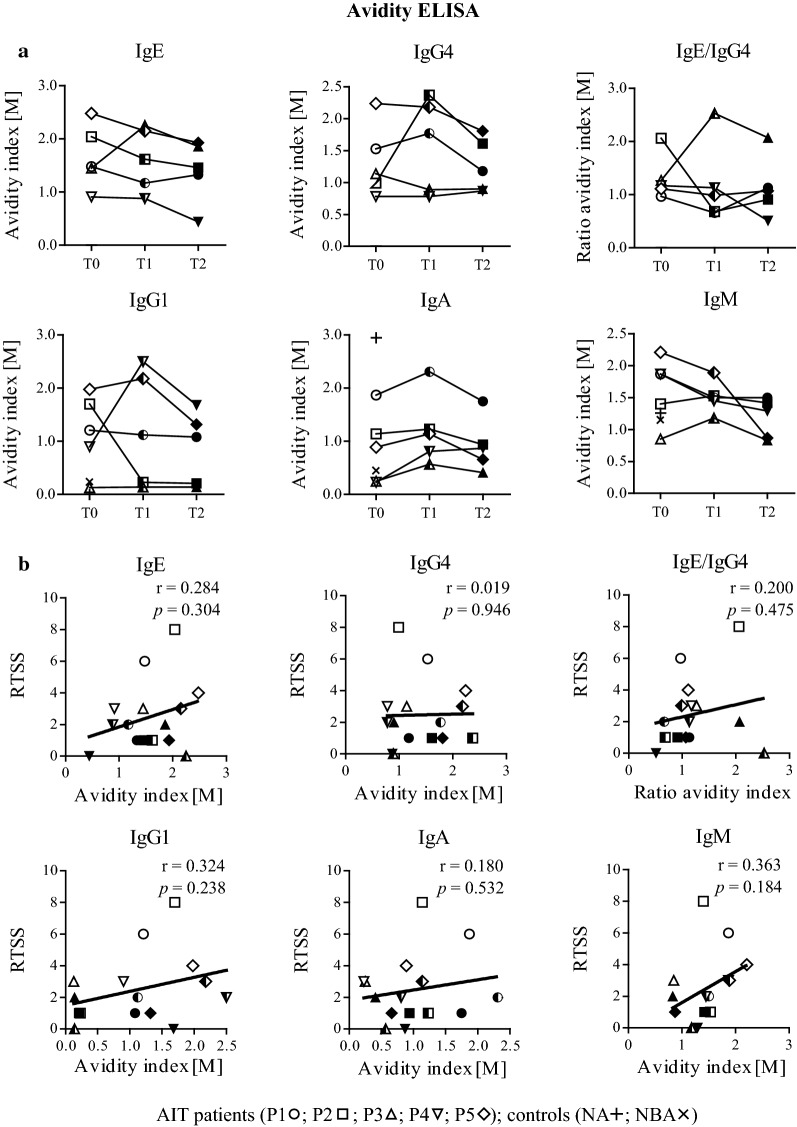
Bet v 1-specific serum antibody avidities during AIT and in control sera analyzed by ELISA. **a** Therapy-related changes in antibody subclass avidity indices. Statistics were performed with Friedman and Dunn’s post test. **b** Correlation of antibody avidity indices with RTSS. Serum samples were obtained at three different time points (T0, open; T1, semi-filled; T2, filled symbols)

We used ELISA to measure avidity indices of Bet v 1-specific antibody subclasses and calculated these as the NaSCN-concentration at which 50% of the bound antibodies were eluted off (Fig. 5, Additional file 5). Bet v 1-specific IgE avidity steadily declined in all patients except for P3, who showed an initial increase followed by a decrease at T2 (Fig. 5a). IgG4 avidity increased in three patients but declined again at T2, whereas it remained more or less in the same range for P3 and P4. Four patients showed constant or decreased IgE/IgG4 avidity index ratios while a strong increase was observed for P3. Therapy-related changes in IgG1 and IgM avidity were heterogeneous and patient-dependent while IgA avidity indices increased slightly but returned to baseline indices. None of the determined antibody avidities nor IgE/IgG4 ratios significantly correlated with RTSS (Fig. 5b). IgA and IgM avidity indices of control sera were similar to those of AIT patients, while due to the lack of IgE and IgG4 values could not be determined for these subclasses (Additional file 5).

### Birch pollen AIT induces blocking antibodies against Bet v 1 in all patients

**Fig. 6:**
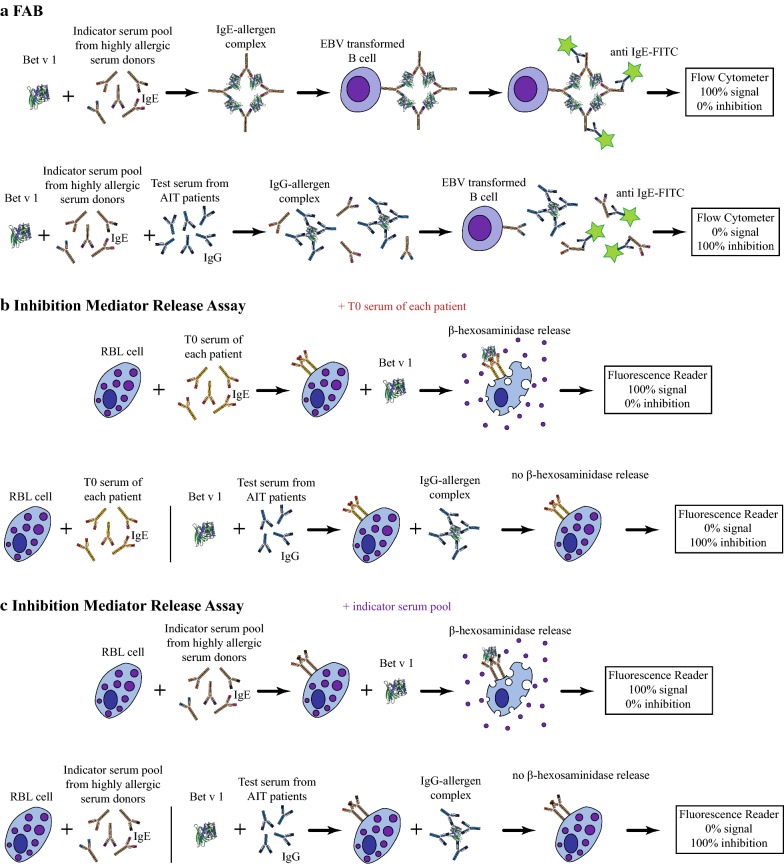
Graphical illustration of assays used for measuring the AIT-induced capacity of antibodies to prevent IgE-Bet v 1 binding. FAB (**a**) and inhibition mediator release assays with T0 serum of each patient (**b**) or indicator serum pool (**c**) used as reference for therapy-induced antibodies to inhibit IgE-allergen binding

**Fig. 7:**
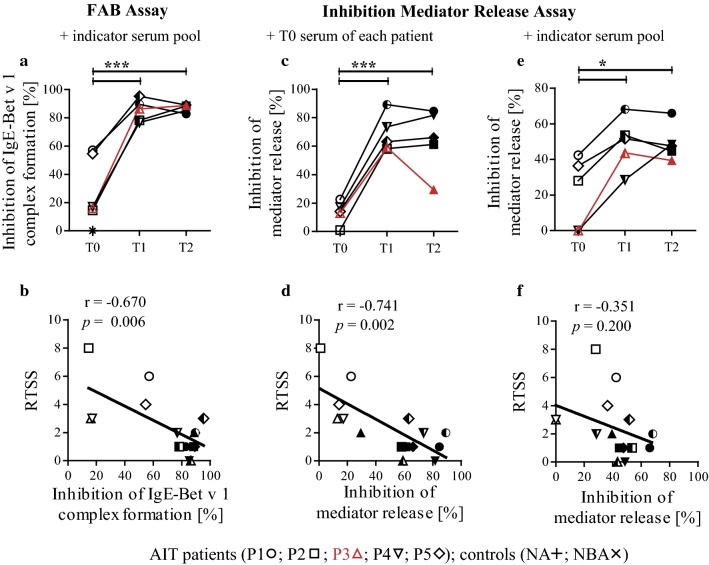
Inhibition of IgE-Bet v 1 binding during AIT and in control sera. Inhibition assays and correlation of complex destruction with RTSS are given for FAB (**a**, **b**), inhibition mediator release assays with T0 serum of each patient (**c**, **d**) and indicator serum pool (**e**, **f**) used as reference for therapy-induced antibodies to inhibit IgE-allergen binding. Statistics were performed with Friedman test and Dunn’s post test. *p < 0.05, ***p < 0.001. Serum of AIT patients (P1–P5) were obtained at three different time points (T0, open; T1, semi-filled; T2, filled symbols)

Next, we performed FAB assays to study the capacity of AIT-induced antibodies to prevent Bet v 1-IgE complex formation in an indicator serum pool containing high amounts of IgE (Fig. 6a). Whereas blocking capacity of antibodies was low at T0, it increased and remained at similar high levels at T2 (Fig. 7a) in three out of five patients. Serum samples of P1 and P5 already revealed an enhanced capacity to block IgE-Bet v 1 complex formation before treatment, which was further increased during AIT. Blocking capacities of antibodies significantly correlated with RTSS (r = − 0.670, p = 0.006) (Fig. 7b). Correlations of blocking capacity and previously determined antibody levels revealed significant values for IgG4 and IgE/IgG4 ratios (Additional file 6). Results of the FAB assay indicate persistent blocking antibody induction in all patients which was able to compete with allergen-IgE complex formation of the indicator serum pool. In contrast, no blocking activity was detectable for both control sera (Fig. 7a).

### AIT does not induce sufficient blocking antibodies in every patient

Inhibition mediator release assays were performed investigating the combined effect of antibody avidity, epitope specificity, and considering the subsequent functional consequence in terms of mediator release. Thereby, RBL cells were passively sensitized with IgE from T0 serum samples (Fig. 6b) or indicator serum pool (Fig. 6c). IgE heat-inactivated T0, T1, and T2 sera were pre-incubated with Bet v 1 and subsequently added to the sensitized cells. ELISA confirmed IgE heat-inactivation, showing lack of reactivity for IgE but not for other antibody subclasses (Additional file 7). β-hexosaminidase release was determined and results expressed as percentage of inhibition of the individual patient.

Whereas inhibition at the beginning of therapy was low, it was significantly higher at T1 for all patients and remained at similar levels for four out of five patients (Fig. 7c, d; Additional file 8). For P3, the inhibition capacity induced at T1 had clearly decreased again at T2, indicating a loss of induced IgG to hinder receptor cross-linking by allergen binding. We did not observe this phenomenon in assays when using an indicator serum pool as reference for FAB assay or for passive sensitization in the inhibition mediator release assays (Fig. 7e, f; Additional file 9). Results of those indicator serum pool-dependent experiments significantly correlated with each other (r = 0.815, p < 0.001). Whereas FAB assay results correlated with therapy outcome, inhibition mediator release results using the indicator serum pool for passive sensitization of the cells did not (Fig. 7). Analogous to FAB assays, correlations of inhibition of IgE-Bet v 1 binding and previously determined antibody levels revealed significant values for IgG4 and IgE/IgG4 ratios using indicator serum pool (Additional file 9B). Notably, inhibition of mediator release values with individual T0 sera for sensitization of cells strongly correlated with clinical treatment outcome (r = − 0.741, p = 0.002) (Fig. 7d). Results of the inhibition mediator release assay with patients’ T0 sera revealed a significant correlation with IgG4 levels, whereas no association was seen for other antibody subclasses or IgE/IgG4 ratios (Additional file 8B). In general, inhibition mediator release studies using patients’ T0 sera revealed highest correlation with clinical parameters. Moreover, unlike in FAB assays, loss of antibody blocking function reflected by aggravation of allergy symptoms could be demonstrated for P3.

## Conclusions

In this study, we fully characterized serum antibody profiles during birch pollen AIT and monitored antibody blocking and immunotherapy-related changes. We thereby intended to uncover parameters influencing blocking capacity to evaluate AIT progress. NPTs were performed to obtain measurable parameters for therapy outcome. Clinical results after 1 year AIT demonstrated beneficial treatment for four subjects, whereas one patient showed a worsening in clinical parameters. Our study clearly focused on monitoring antibody responses during the first year of therapy rather than testing the clinical efficacy of a birch pollen AIT product. For this and ethical reasons which would not justify the application of a placebo, this preliminary antibody monitoring study was conducted within the setting of routine birch pollen AIT in the clinics.

To obtain an initial overview of antibody subclass reactivity towards birch pollen extract, we performed immunoblot assays. Changes in profiles during AIT mostly affected IgE and IgG reactivity to Bet v 1. De novo IgE to Bet v 2 and potentially Bet v 6, Bet v 4, Bet v 7, and Bet v 3 was induced only in P4. After 1 year, novel IgG4 antibodies against Bet v 1 and other proteins were detected in all patients. IgA and IgM did not present relevant changes in antibody reactivity, but control sera generally showed a more complex profile towards those antibody subclasses.

To track Bet v 1-related changes of antibody subclass levels, we performed ELISA experiments. We detected an initial increase in Bet v 1-specific IgE accompanied by an increase in IgG4, which was however lacking in P3. This in turn resulted in an unaltered IgE/IgG4 ratio, while all other patients presented a significantly decreased ratio during AIT. Analogous to previous studies, none of the antibody subclass levels correlated with treatment outcome [10, 11, 22, 35, 36]. In contrast to other investigations, the IgE/IgG4 ratio did not present a useful clinical marker in our study cohort [15, 16]. For antibody quantification, titer determination using ELISA is superior to immunoblots where, due to denaturation and reduction, epitopes might be destroyed as was observed for IgA and IgM. IgE titers correlated well with ImmunoCAP measurements and IgE functionality was verified by mediator release assays. Since not only antibody levels but also avidity might change during AIT [24–27] and, hence influence therapy outcome, we investigated the combined effect of antibody levels and affinities. Compared to antibody titers, Bet v 1-specific IgE and IgG4 avidities showed different patterns lacking typical therapy-induced changes. However, regarding IgE/IgG4 avidity ratios, P3 presented an unusual pattern completely distinct from the other patients.

To additionally address epitope specificities of AIT-induced antibodies, FAB and inhibition mediator release assays were performed. FAB assays revealed that all patients developed blocking antibodies against Bet v 1 during AIT. P2 and P5 had pre-existing Bet v 1-specific IgG but those were only partially able to block IgE-allergen binding. The blocking effect developed during AIT suggests a change in epitope recognition [27]. Similar to other studies, blocking capacities significantly correlated with clinical efficacy [18, 33, 37, 38]. On a patient-by-patient basis, a blocking effect was also detected for P3 after 1 year of treatment suggesting a pitfall of the FAB assay, as clinical parameters for this patient were worsening. This might be due to the indicator serum pool consisting of a variety of unknown IgE epitope specificities—eventually distinct from those of the patient under investigation.

Based on a modified protocol, we performed inhibition mediator release assays to monitor the AIT-induced blocking antibody responses towards the patients’ own IgE epitope specificities and to measure the functional consequence of IgE-allergen binding for mediator release. We showed that low mediator release inhibition before therapy was due to no or low IgG titers. Inhibition significantly increased during therapy revealing that AIT-induced IgG gained the ability to block IgE-specific epitopes [22, 38, 39]. Even though blocking capacity and clinical features of P3 looked promising at T1, a symptom worsening was observed after 1 year, which was clearly reflected by decreased inhibition mediator release at T2. These results seem plausible, as this patient also showed a clear increase in IgE/IgG4 avidity ratios in contrast to the other patients. Potentially, novel IgG epitope specificities developed in the later stage of AIT. Notably, these results were not observed in the FAB assay nor in a FAB-based inhibition mediator release assay. In the latter, we simulated the influence of the broad and potentially distinct IgE epitope specificities in the indicator serum pool on the inhibition of mediator release. This finding clearly demonstrates the necessity of patient-tailored analyses, unequivocally investigating the blocking effect towards the patient’s own IgE epitopes. Notably, although FAB assay results correlated with therapy outcome, FAB based inhibition mediator release results obtained from assays using externally sourced patient IgE for passive sensitization did not. This demonstrates the direct and immense impact of patient IgE from distinct highly allergic individuals in mediator release assays, since here a functional consequence of receptor cross-linking is recorded, which is not considered in FAB assay. Inhibition mediator release results utilizing patient’s own IgE for passive sensitization exhibited a highly significant correlation with the clinical outcome, which was even superior to that obtained with the FAB assay.

The use of patient-tailored inhibition mediator release assays has so far been reported in two studies [22, 39]; however, data was not linked with the clinical outcome. Our study is the first to provide a comprehensive picture of changes in serum antibody responses and their relation to the clinical outcome of AIT. We suggest that IgE and IgG antibodies are key players in determining therapeutic responsiveness. Induced IgG blocking activity is a consequence of both avidity and steric hindrance due to similar IgE and IgG epitope specificities. Only a precise look at all features and their combinations enables illustration of the desired blocking effect indicative of successful therapy. Considering all aspects of this pilot study, we conclude that inhibition mediator release assays using patients’ IgE represents a highly suitable and patient-tailored method to monitor the clinical relevance of blocking antibodies. Further clinical studies following the recently published EAACI guidelines of 3 years AIT treatment and a 2-year follow up should be performed to evaluate our preliminary findings in larger cohorts [40].

## Additional files

**Additional file 1.** Table of patients’ birch pollen allergen antibody titer and total IgE determined by ImmunoCAP.

**Additional file 2.** Table of Bet v 1-specific serum antibody subclass titer.

**Additional file 3.** Correlation of ImmunoCAP values with antibody titer, RTSS and mediator release.

**Additional file 4.** Mediator release curves of RBL-2H3 cells during birch pollen AIT and correlation with antibody titer.

**Additional file 5.** Table of Bet v 1-specific serum antibody subclass avidity indices.

**Additional file 6.** Correlation of Bet v 1-specific serum antibody titer with percent inhibition of IgE-Bet v 1 complex formation measured by FAB assay.

**Additional file 7.** Reactivity of serum antibody subclasses before and after heat inactivation.

**Additional file 8.** Inhibition mediator release curves and correlation with antibody titer using cells passively sensitized with T0 sera.

**Additional file 9.** Inhibition mediator release curves and correlation with antibody titer using cells passively sensitized with indicator serum pool.
