# Supplementary material for: Does clinical outcome of birch pollen immunotherapy relate to induction of blocking antibodies preventing IgE from allergen binding? A pilot study monitoring responses during first year of AIT
Source: Clin Transl Allergy. 2018 Oct 8;8:39. doi: 10.1186/s13601-018-0226-7 (PMC6174570; doi:10.1186/s13601-018-0226-7)
Supplement: Supplementary file 1 — Additional file 1. Table of patients’ birch pollen allergen antibody titer and total IgE determined by ImmunoCAP. [file 13601_2018_226_MOESM1_ESM.pdf]

Additional file 1. ImmunoCAP analyses of AIT patients' sera.

**Patients**	**Bet v 1-specific IgE [kUA/l]**			**Bet v 2-specific IgE [kUA/l]**			**Bet v 4-specific IgE [kUA/l]**			**Total IgE [kU/l]**			**Bet v 1-specific IgG4 [mgA/l]**		
	T0	T1	T2	T0	T1	T2	T0	T1	T2	T0	T1	T2	T0	T1	T2
P1	40.2	42.7	19.8	<0.1	<0.1	<0.1	<0.01	<0.01	<0.01	90.5	80.3	49.9	0.3	0.6	4.3
P2	4.4	5.8	4.8	<0.01	<0.1	<0.1	<0.01	<0.01	<0.01	23.2	28.7	22.0	0.2	1.9	3.3
P3	26.3	53.3	38.0	<0.1	<0.1	<0.1	<0.01	<0.01	<0.01	188.0	203.0	156.0	0.7	3.0	3.5
P4	51.1	159.0	81.3	<0.1	0.1	0.17	<0.01	<0.01	<0.01	292.0	463.0	488.0	<0.1	2.9	4.3
P5	10.3	17.5	10.0	<0.1	<0.1	<0.1	<0.1	<0.1	<0.1	37.1	51.9	40.5	1.3	5.7	4.6

P1–5, AIT patients; T0, before AIT; T1, two weeks after reaching the maintenance dose; T2, one year after starting AIT
