# Supplementary material for: Does clinical outcome of birch pollen immunotherapy relate to induction of blocking antibodies preventing IgE from allergen binding? A pilot study monitoring responses during first year of AIT
Source: Clin Transl Allergy. 2018 Oct 8;8:39. doi: 10.1186/s13601-018-0226-7 (PMC6174570; doi:10.1186/s13601-018-0226-7)
Supplement: Supplementary file 2 — Additional file 2. Table of Bet v 1-specific serum antibody subclass titer. [file 13601_2018_226_MOESM2_ESM.pdf]

Additional file 2. Bet v 1-specific serum antibody titer.

Serum	IgE titer			IgG4 titer			IgG1 titer			IgA titer			IgM titer			Ratio titer IgE/IgG4		
	T0	T1	T2	T0	T1	T2	T0	T1	T2	T0	T1	T2	T0	T1	T2	T0	T1	T2
P1	2926	2377	1737	597	1506	13854	1258	5461	6443	21806	15863	16514	23680	22720	20267	4.90	1.58	0.13
P2	203	226	208	200	5369	7502	162	441	450	9542	10705	9309	6991	8172	4726	1.02	0.04	0.03
P3	951	2286	1493	899	2198	1419	96	94	100	17371	19017	16823	4928	6080	3392	1.06	1.04	1.05
P4	1327	1967	1253	105	3760	7320	2400	1653	2187	7782	13824	11469	26880	28800	23253	12.64	0.52	0.17
P5	231	284	251	597	14440	15520	4538	7447	10938	3482	5427	5325	16640	17067	17280	0.39	0.02	0.02
NA	3			0			37			8611			11324			id		
NBA	7			14			284			5440			7680			0.50		
IND	14938			527			762			7694			30927			28.35		

P1–5, patients receiving birch pollen AIT; NA, non-allergic serum donor; NBA, non birch allergic serum donor; IND, indicator serum pool; T0, before AIT; T1, two weeks after reaching the maintenance dose; T2, one year after starting AIT; id, indeterminable
