# Supplementary material for: Does clinical outcome of birch pollen immunotherapy relate to induction of blocking antibodies preventing IgE from allergen binding? A pilot study monitoring responses during first year of AIT
Source: Clin Transl Allergy. 2018 Oct 8;8:39. doi: 10.1186/s13601-018-0226-7 (PMC6174570; doi:10.1186/s13601-018-0226-7)
Supplement: Supplementary file 3 — Additional file 3. Correlation of ImmunoCAP values with antibody titer, RTSS and mediator release. [file 13601_2018_226_MOESM3_ESM.pdf]

Correlation ImmunoCAP

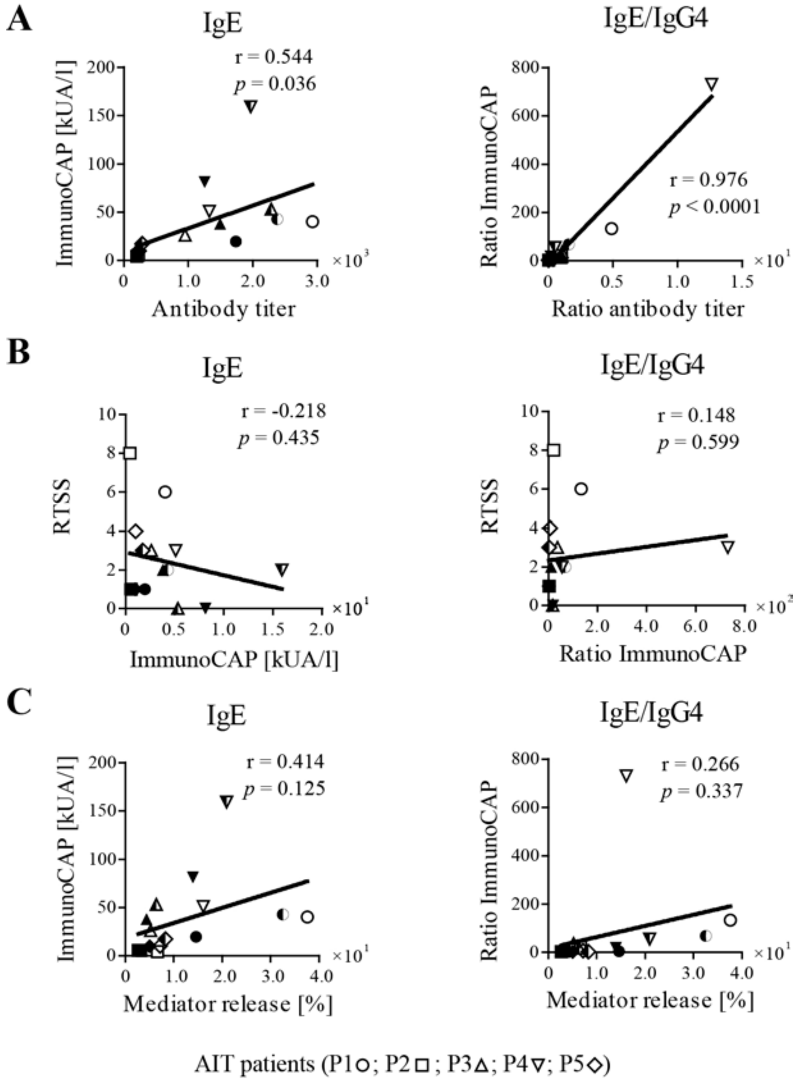

Additional file 3. Correlation of Bet v 1-specific serum IgE levels and IgE/IgG4 ratios measured by ImmunoCAP with levels determined by ELISA (A), RTSS (B), and percent mediator release triggered by a concentration of 10 ng/ml Bet v 1 (C). Serum samples were obtained at three different time points (T0, open; T1, semi-filled; T2, filled symbols).
