# Supplementary material for: Does clinical outcome of birch pollen immunotherapy relate to induction of blocking antibodies preventing IgE from allergen binding? A pilot study monitoring responses during first year of AIT
Source: Clin Transl Allergy. 2018 Oct 8;8:39. doi: 10.1186/s13601-018-0226-7 (PMC6174570; doi:10.1186/s13601-018-0226-7)
Supplement: Supplementary file 4 — Additional file 4. Mediator release curves of RBL-2H3 cells during birch pollen AIT and correlation with antibody titer. [file 13601_2018_226_MOESM4_ESM.pdf]

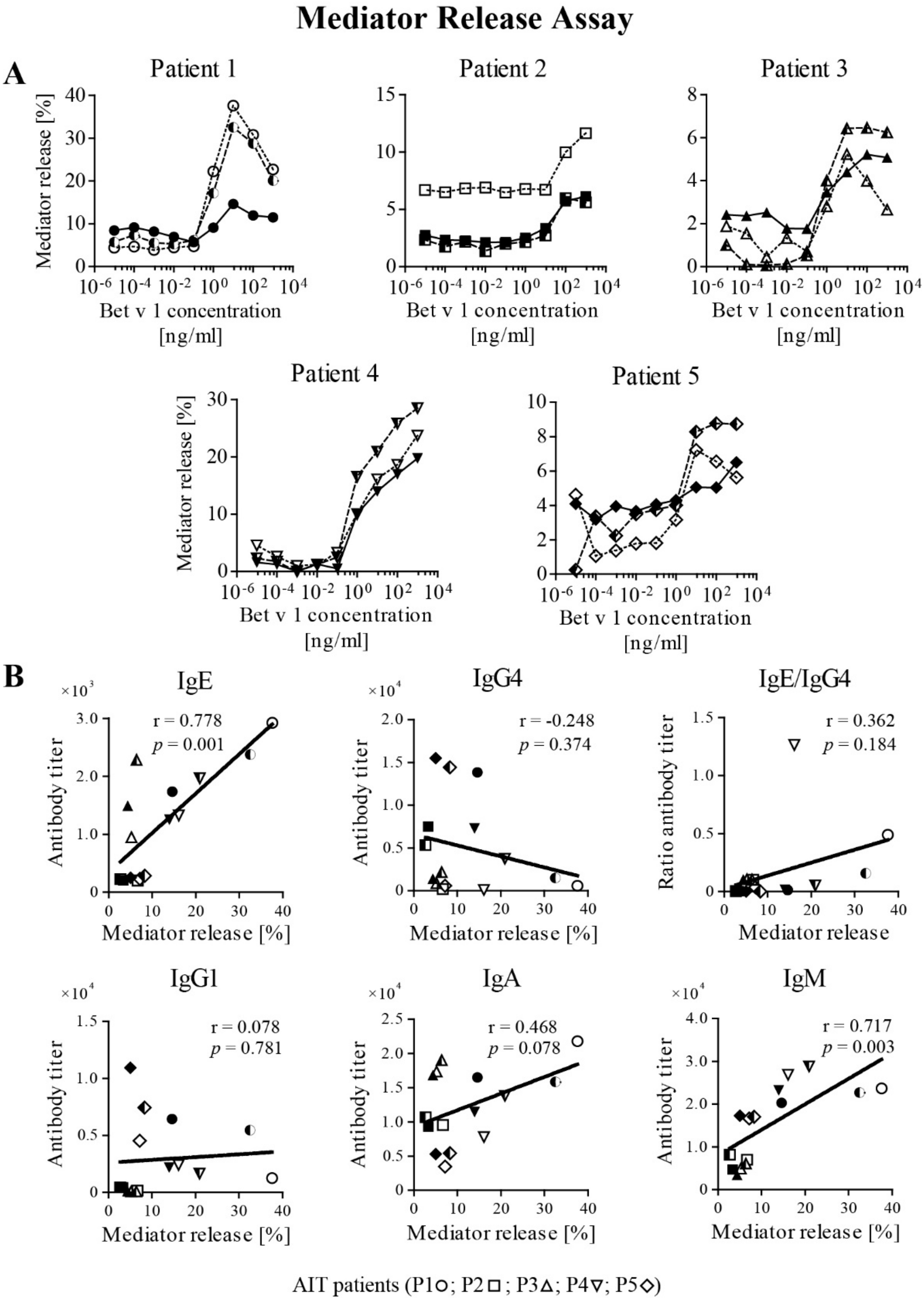

Additional file 4. Mediator release of RBL-2H3 cells during AIT. For each patient the percentage of β-hexosaminidase release was determined at different Bet v 1 concentrations with sera obtained at three different time points (T0, open; T1, semi-filled; T2, filled symbols) of AIT (A). Correlation of Bet v 1-specific serum antibody titer measured by ELISA with mediator release triggered by a concentration of 10 ng/ml Bet v 1 (B).
