# Supplementary material for: Does clinical outcome of birch pollen immunotherapy relate to induction of blocking antibodies preventing IgE from allergen binding? A pilot study monitoring responses during first year of AIT
Source: Clin Transl Allergy. 2018 Oct 8;8:39. doi: 10.1186/s13601-018-0226-7 (PMC6174570; doi:10.1186/s13601-018-0226-7)
Supplement: Supplementary file 5 — Additional file 5. Table of Bet v 1-specific serum antibody subclass avidity indices. [file 13601_2018_226_MOESM5_ESM.pdf]

Additional file 5. Bet v 1-specific serum antibody avidity indices.

	**IgE avidity index [M]**			**IgG4 avidity index [M]**			**IgG1 avidity index [M]**			**IgA avidity index [M]**			**IgM avidity index [M]**			**Ratio avidity indices IgE/IgG4**		
	T0	T1	T2	T0	T1	T2	T0	T1	T2	T0	T1	T2	T0	T1	T2	T0	T1	T2
P1	1.48	1.17	1.33	1.53	1.77	1.18	1.21	1.12	1.08	1.87	2.31	1.75	1.87	1.50	1.50	0.97	0.66	1.13
P2	2.04	1.62	1.46	0.99	2.37	1.61	1.70	0.23	0.21	1.14	1.23	0.94	1.40	1.53	1.42	2.06	0.68	0.91
P3	1.45	2.25	1.86	1.14	0.89	0.9	0.13	0.14	0.14	0.25	0.57	0.41	0.85	1.18	0.83	1.27	2.53	2.07
P4	0.91	0.88	0.44	0.78	0.78	0.87	0.90	2.50	1.68	0.23	0.81	0.87	1.87	1.45	1.29	1.17	1.13	0.51
P5	2.48	2.15	1.93	2.24	2.18	1.81	1.98	2.18	1.32	0.89	1.14	0.66	2.21	1.89	0.87	1.11	0.99	1.07
NA	id			id			id			2.95			1.26			id		
NBA	id			id			0.24			0.45			1.15			id		
IND	1.20			0.60			2.23			1.99			2.81			0.54		

P1–5, patients receiving birch pollen AIT; NA, non-allergic serum donor; NBA, non birch allergic serum donor; IND, indicator serum pool; [M], molar concentration; T0, before AIT; T1, two weeks after reaching the maintenance dose; T2, one year after starting AIT; id, indeterminable
