# Supplementary material for: Does clinical outcome of birch pollen immunotherapy relate to induction of blocking antibodies preventing IgE from allergen binding? A pilot study monitoring responses during first year of AIT
Source: Clin Transl Allergy. 2018 Oct 8;8:39. doi: 10.1186/s13601-018-0226-7 (PMC6174570; doi:10.1186/s13601-018-0226-7)
Supplement: Supplementary file 6 — Additional file 6. Correlation of Bet v 1-specific serum antibody titer with percent inhibition of IgE-Bet v 1 complex formation measured by FAB assay. [file 13601_2018_226_MOESM6_ESM.pdf]

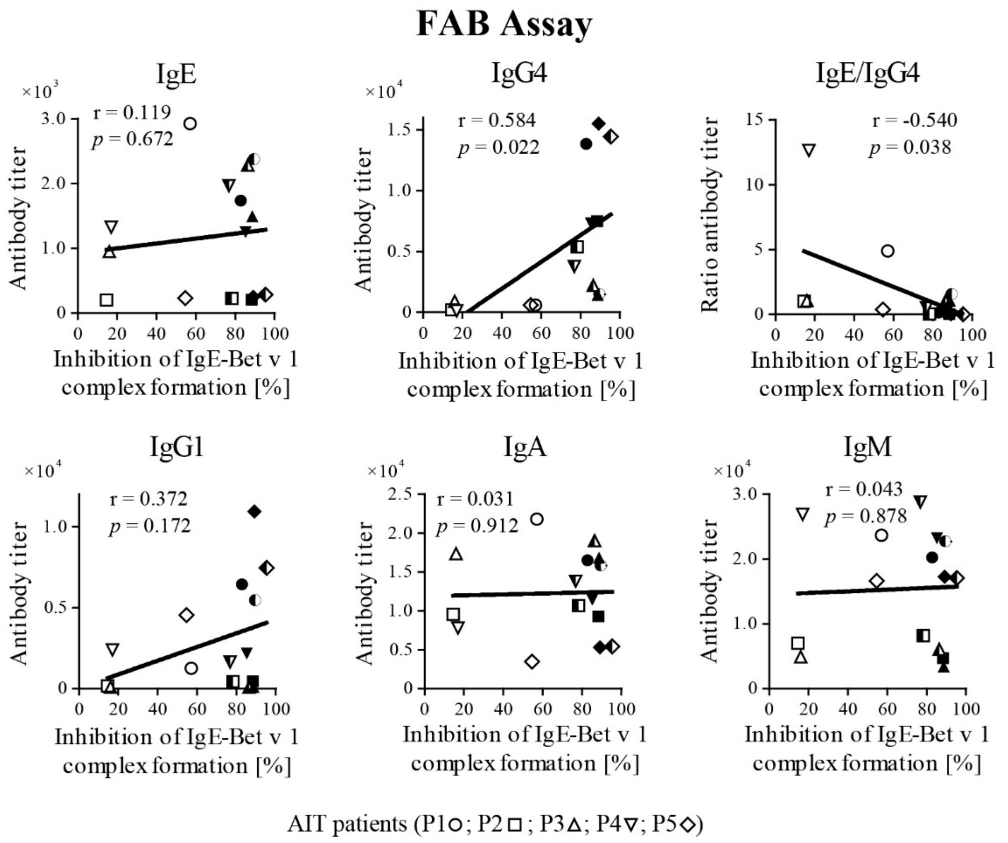

Additional file 6. Correlation of Bet v 1-specific serum antibody titer measured by ELISA with percent inhibition of IgE-Bet v 1 complex formation measured by FAB assay. Serum samples were obtained at three different time points (T0, open; T1, semi-filled; T2, filled symbols) and correlated with each other.
