# Supplementary material for: Does clinical outcome of birch pollen immunotherapy relate to induction of blocking antibodies preventing IgE from allergen binding? A pilot study monitoring responses during first year of AIT
Source: Clin Transl Allergy. 2018 Oct 8;8:39. doi: 10.1186/s13601-018-0226-7 (PMC6174570; doi:10.1186/s13601-018-0226-7)
Supplement: Supplementary file 7 — Additional file 7. Reactivity of serum antibody subclasses before and after heat inactivation. [file 13601_2018_226_MOESM7_ESM.pdf]

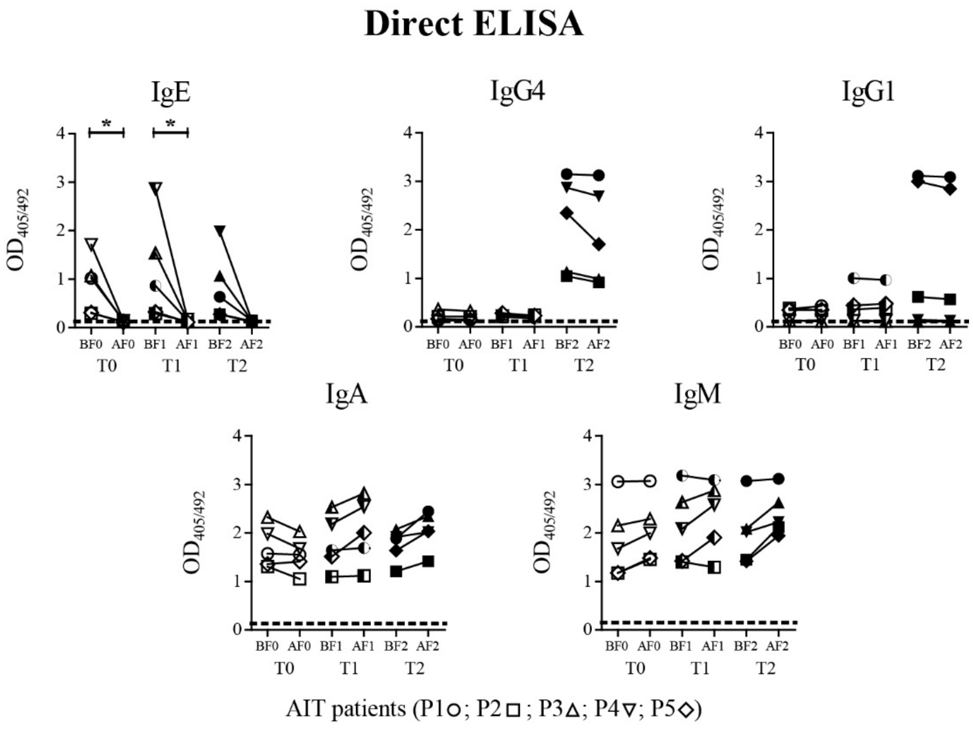

Additional file 7. Reactivity of serum antibody subclasses before (BF) and after (AF) heat inactivation. The $OD_{405/492}$ of the serum samples was obtained at three different time points of AIT (T0, open; T1, semi-filled; T2, filled symbols). The limit of detection defined as three times standard deviation of the buffer control was used as cutoff value.
