# Supplementary material for: Does clinical outcome of birch pollen immunotherapy relate to induction of blocking antibodies preventing IgE from allergen binding? A pilot study monitoring responses during first year of AIT
Source: Clin Transl Allergy. 2018 Oct 8;8:39. doi: 10.1186/s13601-018-0226-7 (PMC6174570; doi:10.1186/s13601-018-0226-7)
Supplement: Supplementary file 8 — Additional file 8. Inhibition mediator release curves and correlation with antibody titer using cells passively sensitized with T0 sera. [file 13601_2018_226_MOESM8_ESM.pdf]

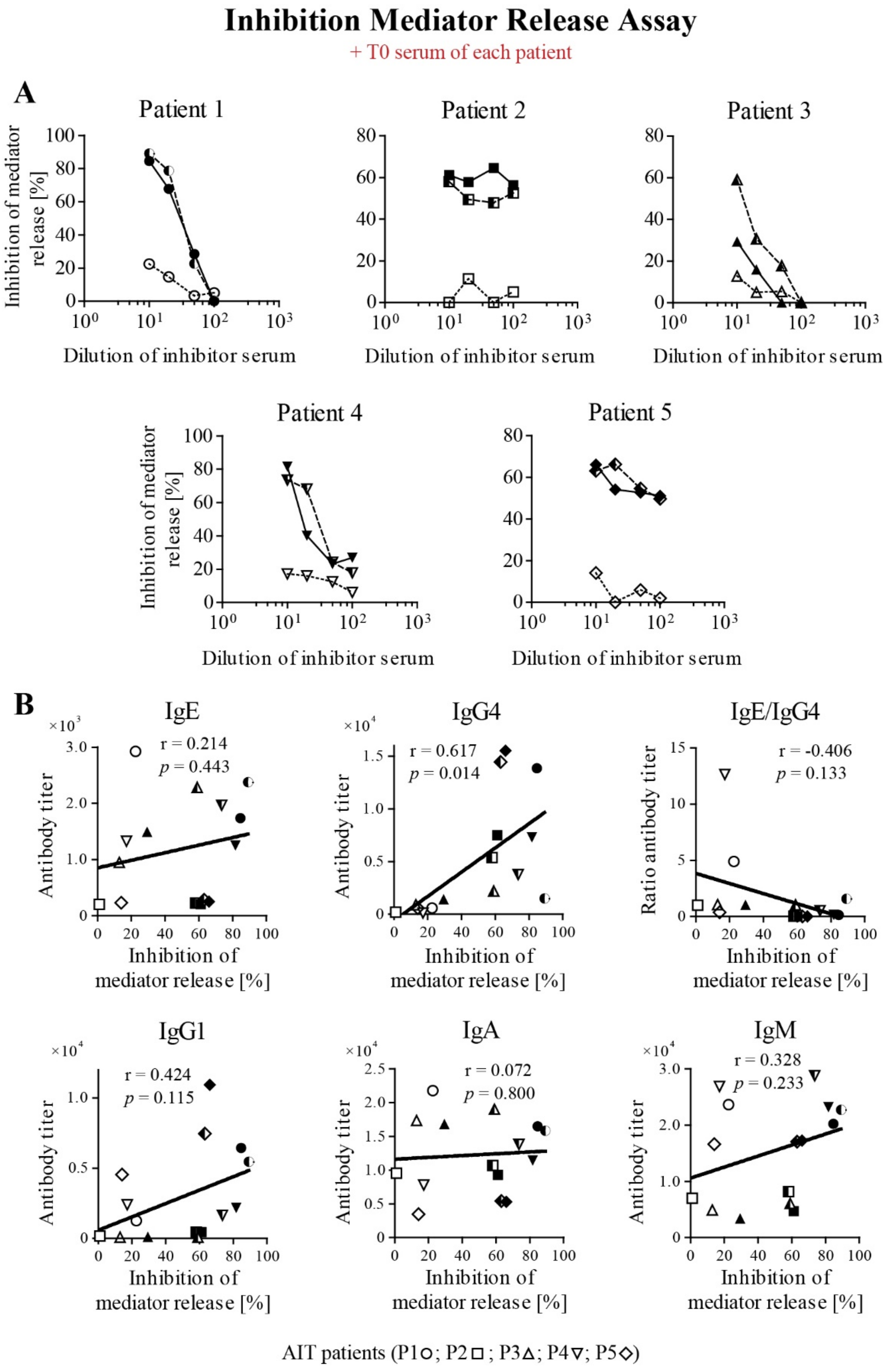

Additional file 8. Inhibition of mediator release by induced antibodies during AIT. Cells were passively sensitized with T0 sera of each AIT patient and inhibited with different dilutions of serum samples from three AIT time points (T0, open; T1, semi-filled; T2, filled symbols) (A). Correlation of Bet v 1-specific antibody titer measured by ELISA with inhibition mediator release measured by assays using T0 sera of each patient for sensitizing the cells (B).
