# Supplementary material for: Does clinical outcome of birch pollen immunotherapy relate to induction of blocking antibodies preventing IgE from allergen binding? A pilot study monitoring responses during first year of AIT
Source: Clin Transl Allergy. 2018 Oct 8;8:39. doi: 10.1186/s13601-018-0226-7 (PMC6174570; doi:10.1186/s13601-018-0226-7)
Supplement: Supplementary file 9 — Additional file 9. Inhibition mediator release curves and correlation with antibody titer using cells passively sensitized with indicator serum pool. [file 13601_2018_226_MOESM9_ESM.pdf]

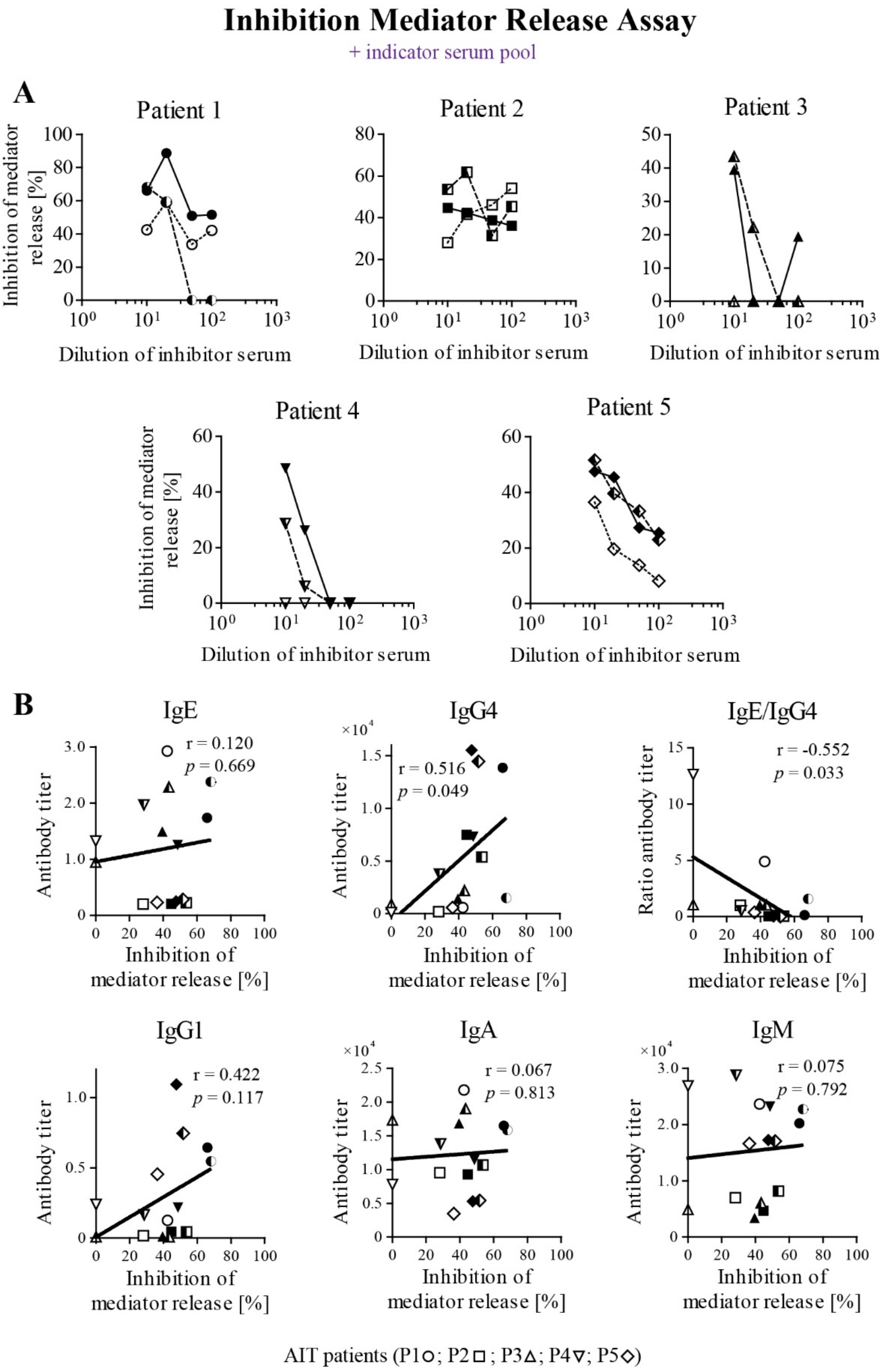

Additional file 9. Inhibition of mediator release by induced antibodies during AIT. Cells were passively sensitized with a FAB-based indicator serum pool and inhibited with different dilutions of serum samples from three AIT time points (T0, open; T1, semi-filled; T2, filled symbols) (A). Correlation of Bet v 1-specific antibody titer measured by ELISA with inhibition mediator release measured by assays using an indicator serum pool for sensitizing the cells (B).
